# Supplementary material for: Difficult biliary cannulation among patients with compensated liver cirrhosis: predictors and impact on complications
Source: Sci Rep. 2026 Mar 19;16:9533. doi: 10.1038/s41598-026-41040-1 (PMC13004906; doi:10.1038/s41598-026-41040-1)
Supplement: Supplementary file 1 — Supplementary Material 1 [file 41598_2026_41040_MOESM1_ESM.docx]

**Difficult biliary cannulation among patients with compensated Liver cirrhosis: Predictors and impact on complications**

**Mahmoud A. Elkerdawy, Msc**

Hepatology, Gastroenterology and Infectious Diseases Department, Faculty of Medicine, Kafrelsheikh University, KAFR ELSHIEKH, 33516, Egypt. Email: [Dr.Kerdawy10@gmail.com](mailto:Dr.Kerdawy10@gmail.com), ORCID: 0000-0003-2259-3209. ‎

**Aya Mohammed Mahros, MD**

Hepatology, Gastroenterology and Infectious Diseases Department, Faculty of Medicine, Kafrelsheikh University, KAFR ELSHIEKH, 33516, Egypt.**Email:** [**yoye_85@hotmail.com**](mailto:yoye_85@hotmail.com)

**Mohamed H Emara, MD**

Hepatology, Gastroenterology and Infectious Diseases Department, Faculty of Medicine, Kafrelsheikh University, KAFR ELSHIEKH, 33516, Egypt. Email: emara_20007@yahoo.com, ORCID: 0000-0002-1504-7851

**Mohammed Hussien Ahmed, MD**

Hepatology, Gastroenterology and Infectious Diseases Department, Faculty of Medicine, Kafrelsheikh University, KAFR ELSHIEKH, 33516, Egypt.Email: [**dr.mm63@yahoo.com**](mailto:dr.mm63@yahoo.com)

**Ahmed Ebeed, MD**

Diagnostic and Interventional Radiology Department, Faculty of Medicine, Kafrelsheikh University, KAFR ELSHIEKH, 33516, Egypt, Email: [drahmedebeed@yahoo.com](mailto:drahmedebeed@yahoo.com)

**Hassan E Elbatae, MD**

Hepatology, Gastroenterology and Infectious Diseases Department, Faculty of Medicine, Kafrelsheikh University, KAFR ELSHIEKH, 33516, Egypt. Email: [hassanelbatae_68@yahoo.com](mailto:hassanelbatae_68@yahoo.com)

**Corresponding author:**

**Mohamed H Emara, MD**

Hepatology, Gastroenterology and Infectious Diseases Department, Faculty of Medicine, Kafrelsheikh University, KAFR ELSHIEKH, 33516, Egypt. Email: emara_20007@yahoo.com, Tel. +201002724482

**Short title:** Difficult biliary cannulation and compensated Liver cirrhosis.

**ABSTRACT**

**Background:** This study investigated prospectively how frequent is difficult biliary cannulation (DBC) among patients with compensated liver cirrhosis and how it will impact the ERCP related complications.

**Patients and methods:** Over a period of 2-years, patients with compensated liver cirrhosis undergoing their first time ERCP with naive papilla were evaluated by history, clinical examination, investigations, imaging and ERCP and followed up for any adverse events.

**Results:** Out of 131 compensated cirrhotic patients; 127 were successfully cannulated and included in the final analysis and divided into easy (n=72) and difficult (n=55) cannulation groups. The mean age of the studied cases was 51.86±11.98 years (19-78) with male predominance (51.2% versus 48.8%). The most frequent indication for ERCP was calcular obstructive jaundice. DBC was reported among 45.5% of cases. DBC was associated with 12.6% complication rate of them 8.7% had pancreatitis and 3.9% had intraoperative minor bleeding. Older age, type 2 and 3 papillae, duodenal diverticulum and precut sphincterotomy were predictors of DBC.

**Conclusion**: DBC is not uncommon among compensated cirrhotic patients experiencing their first time ERCP. Because DBC is associated with ERCP complications, its predictors should be considered especially the type of papilla, the presence of duodenal diverticulum and assisted cannulation techniques.

**Key Words:** Endoscopic retrograde cholangiopancreatography; Difficult biliary cannulation; Liver cirrhosis; Papilla.

**INTRODUCTION**

The role of Endoscopic retrograde cholangiopancreatography (ERCP) has evolved from a mere diagnostic to a mainly therapeutic intervention because of the achievements in the imaging methods, including magnetic resonance imaging (MRI), magnetic resonance cholangiopancreatography (MRCP) and endoscopic ultrasound (EUS). Although considered minimally invasive, ERCP procedure is associated with adverse events which can lead to very serious complications [1].

The list of indications for ERCP is not limited to many benign (stone, sludge, cholangitis, sphincter of Oddi dysfunction, pancreatitis) but also to malignant (periampullary tumor, cholangiocellular tumors) diseases of pancreatobiliary tract [2,3]***.*** However, several factors contribute to clinical success and adverse events related to ERCP. These factors, include patient-related, endoscopist-related, and technical parameters. Furthermore, complications related to sedation add to the ERCP associated adverse events [4]. Out of technical parameters, difficult biliary cannulation (DBC) has been shown to increase the risk of ERCP related adverse events [5,6].

Post-ERCP complications are very important causes of morbidity and less frequently mortality [7]. Even with experienced endoscopists, still complications do occur at varying rates after the ERCP procedure. Patients with liver cirrhosis tolerate ERCP differently than other patients. In fact, earlier reports showed that patients with cirrhosis had more bleeding and more severe pancreatitis than low risk subjects [8]**,** furthermore the infectious and inflammatory responses are associated with liver function deterioration [9]. The aim of the current study was to investigate prospectively how frequent is DBC among a cohort of compensated liver cirrhosis patients and how it will impact the ERCP related success and complications.

**PATIENTS AND METHODS**

This study was carried out on patients attending Kafrelsheikh University Hospitals, Egypt during the period from November 2022 to November 2024.

The current study included ERCP naive patients of both sexes aged more than 18 years with a native papilla and compensated liver cirrhosis (diagnosed based on the history, through clinical examination, laboratory investigations, imaging studies, and/or liver biopsy and functionally categorized following Child-Pugh score) when the indications for ERCP were clear and discussed with the patients. The Exclusion criteria comprised previous ERCP, decompensated cirrhosis (Child B, C), postoperative altered anatomy, duodenal and/or biliopancreatic trauma and pregnancy.

***All cases were subjected to:***

1. **Full history taking** including age, sex, occupation, smoking habits, viral hepatitis status, manifestations of hepatic decompensation, variceal interventions and co-morbidities.
2. **Complete clinical examination**
3. **Laboratory tests:** complete blood count, amylase and lipase levels, liver and kidney function tests.
4. **Radiological**: Ultrasound abdomen - MRCP
5. **ERCP indication** was recorded, and Child class/score was calculated.
6. **ERCP Procedure**:

**Before ERCP:** Preanesthetic assessment for all patients was routinely done.

**During ERCP:** All patients received rectal indomethacin immediately before the procedure, besides good hydration with lactated Ringer’s solution during the ERCP.

ERCP procedures were performed by two experienced endoscopists (with an annual ERCP rate more than 400) and two senior fellows in advanced endoscopy (with an experience of over 180 ERCPs performed). The guidewire-assisted technique was used as the primary (standard) biliary cannulation technique. Advanced cannulation techniques were performed following the endoscopist’s decision only in the case of difficult standard cannulation. These comprised freehand needle knife sphincterotomy (cutting from the papillary orifice upwards), needle knife fistulotomy (making a whole above the papillary orifice), and trans-pancreatic biliary sphincterotomy (cutting the septum between pancreatic and common bile duct after pancreatic duct cannulation), the later was considered after the second unintentional pancreatic duct cannulation. Mixed electrosurgical current was used in all cases for papillotomy [10-13].

In all ERCP procedures the duodenoscope was advanced to the papillary region and morphology of the papilla was assessed. Its appearance was classified according to Haraldsson’s classification into: Regular (Type 1), Small sized with pin-point orifice (Type 2), large Protruding or Pendulous (Type 3), and
Creased or Ridged (Type 4) [10]. Presence of duodenal diverticula was documented.

Bile duct cannulation was monitored. The number of intentional contacts with the papilla for attempted cannulation was noted. The time between the first intentional contact and bile duct cannulation confirmed by fluoroscopy was recorded. Unintentional guidewire passages in the main pancreatic duct were recorded. The need for freehand precut sphincterotomy, fistulotomy or transpancreatic septotomy was documented. Difficult cannulation was defined following the criteria established by the European Society for Gastrointestinal Endoscopy (ESGE) clinical guideline as: more than 5 contacts with the papilla while attempting to cannulate; more than 5 min attempting to cannulate following first intentional contact of the papilla; more than one unintended pancreatic duct cannulation or opacification [11].

**After ERCP:** All patients were hospitalized for at least 24 h with clinical assessment, as well as a biochemical panel including complete cell count, C reactive protein levels, pancreatic enzymes, liver and kidney function tests.

Further hospitalization and monitoring relied on the presence or absence of post-ERCP complications. All patients were prospectively followed up following the procedure in the outpatient clinic.

1. **Data for prediction of post-ERCP complications** were recorded including (the methods of cannulation, cannulation attempts and time to successful cannulation, bile duct anatomy, insertion of stents, brush cytology or balloon sphincteroplasty, pancreatic duct cannulation).
2. **Adverse events** including post-ERCP pancreatitis (PEP), bleeding, infection and perforation were defined according to consensus criteria of the American Society of Gastrointestinal Endoscopy (ASGE) [1]. Definition and classification of post-ERCP pancreatitis followed the revised Atlanta classification criteria [12] which is currently preferred than the standard grading following Cotton criteria because it accurately predicts mortality. A cannulation attempt was defined as any repositioning or wedging of the catheter tip or cannulation device in an attempt to cannulate the biliary or pancreatic duct. Sphincterotome cannulations in cases that would aid cannulation (cautery was not applied in this situation; the sphincterotome was used for its improved maneuverability compared to standard cannulas, which do not have a bowing mechanism). Guidewire cannulations in cases in which a guide wire was used to assist in cannulation [1,11,12].

***Statical analysis***

**Sample size**

All patients attending both the outpatient clinic and the endoscopy unit of the Hepatology, Gastroenterology and the Infectious Diseases Department, Kafrelsheikh University Hospital during the study period fulfilling the inclusion criteria were offered to participate in the study.

**End points**

Primary end point of the current study was the ease of biliary cannulation while secondary end points were the short-term adverse events associated with ERCP and predictors of DBC.

**Statistical analysis and data interpretation:**

Data analysis was performed by SPSS software, version 26. Qualitative data were described using number and percent. Quantitative data were described using median (minimum and maximum) for non-normally distributed data and mean± Standard deviation for normally distributed data after testing normality using Kolmogrov-Smirnov test. Significance of the obtained results was judged at the (0.05) level. Chi-Square, Fisher exact test were used to compare qualitative data between groups as appropriate. Mann Whitney U test was used to compare between 2 studied groups for non-normally distributed data. Student t test was used to compare 2 independent groups for normally distributed data. Receiver operating characteristics curve (ROC curve) was used to calculate validity (sensitivity & specificity) of continuous variables with calculation of best cut off point. Binary logistic regression was used to assess the effect of combination of more than 2 independent variables on dichotomous outcome using Enter technique

**RESULTS**

***Base line characteristics***

This cross-sectional study initially focused on 503 patients presented for ERCP during the study period **(Figure 1)**, among them 171 patients were suspected cirrhotics (33.9%); 40 cases were excluded following our inclusion/exclusion criteria while 4 cases with failed biliary cannulation were excluded from the final analysis. Finally, 127 compensated cirrhotic patients with clear indications for ERCP were successfully cannulated and analyzed. Per ease of biliary cannulation and occurrence of ERCP related adverse events they were divided into 2 groups: difficult and easy cannulation groups. The demographic features, and clinical data are shown in **Table 1.**

The mean age of the studied cases was 51.86±11.98 years (19-78) with male predominance (51.2% versus 48.8%). Besides being compensated cirrhotics, patients had other comordidities;30.7% were diabetic, 27.6% were hypertensive, 11.8% had other comorbidities e.g., obesity, nephropathy, lupus and 4.7% had previous surgical history (non-hepatobiliary) while 25.2% of the cohort are currently smokers. Clinically, 81.9% of the patients presented with calcular obstructive jaundice, 15% had biliary pain, 12.6% had confirmed malignancy, 7.9% had cholangitis and 1.6% had pancreatitis.

Apart from age, no statistically significant difference was observed regarding demographic characters, associated comorbidities, and smoking history between both groups. Furthermore, the indications for ERCP and the clinical presentation were comparable in both groups **(Table 1);** calcular obstructive jaundice with its consequences were the most common presentation (n=104, 81.9%). Patients with difficult cannulation tend to be older than easy cannulation group (P=0.014).

***Biliary cannulation methods***

Different cannulation techniques had been used for accessing the biliary system in this study. Guidewire assisted technique was used among 59.1% out of them 56.7% (n=72; 56.9%) were successfully accessed according to the predefined criteria and represents the easy cannulation group while 2.4% failed the criteria although still cannulated by the guidewire without assisted techniques. Other techniques of assisted cannulation comprised precut sphincterotomy, double guide, and fistulotomy in 33.1%, 4.7% and 3.1% respectively. The difficult cannulation group (n=55; 43.3%) included cases of assisted cannulation and the 2.4% of guidewire cannulation. Median cannulation time is 5 minutes ranging from 2 to 23 and median cannulation attempts was 3 ranging from 1 to 8 **(Table 2)**.

***Outcomes of ERCP***

Successful biliary cannulation rate in the current study was 96.9% (127/131). The four cases with failed biliary cannulation were treated with EUS guided drainage because of locally advanced pancreatic cancer in one patient, while another session of ERCP using Rendezvous technique was successful in 3 cases.

The rate of adverse events **(Table 3)** in the current study was 12.6% (16/127). DBC was associated with ERCP complications in the current study. Complications; pancreatitis and bleeding were reported in the DBC group (significant difference; p<0.001 and 0.014, respectively). The incidence of pancreatitis was 8.7% (11/127); most of the cases were mild and discharged from the hospital 1-2 days post procedural, while 3 cases were moderate to severe with longer hospital stay. The bleeding rate was 3.9% (5/127) and all cases had minor bleeding treated conservatively intraoperatively and none had delayed bleeding nor required blood transfusion. Furthermore, no cases of perforation, cholangitis nor death were reported in the current study. Focusing on demographics and clinical data of the complicated cases **(Table 1)**, a statistically significant higher incidence of complications was detected among older age (p= 0.001), smokers (p=0.04) and in the context of other comorbidities including obesity, lupus, and nephropathy (p=0.006) as shown in **Table 1**. Type 1 papilla was the most common type among patients 63.6% (77/121). A statistically significant higher incidence of complications reported among cases with type 2 papilla and 53.3% of duodenal diverticulum cases had complications. Bleeding was higher among cases with papilla type 3 followed by papilla type 4, whereas there was no relation between shape of the papilla and pancreatitis **(Table 3).** During the short-term follow up there were no cases of clinical or laboratory liver function deterioration.

***Difficult cannulation and ERCP Outcomes***

Different endoscopic parameters were studied in relation to the ease of biliary cannulation and occurrence of complications **(Table 2)**. There was a statistically significant difference between cases of DBC and those without regarding; shape of duodenal papilla (p=0.03), the presence of duodenal diverticulum (p=0.018), distal CBD stricture on imaging, use of assisted cannulation methods, cannulation time and cannulation attempts as shown in **Table 2.** However, there was no difference/relation regarding the diameter of the dilated CBD (without stricture), stone size or the presence of pancreatic masses.

Parameters of DBC (assisted cannulation), type 2/3 papilla, presence of duodenal diverticulum, prolonged median cannulation time and higher attempts of cannulation were statistically significantly associated with the occurrence of complications **(Table 2).**

The predictors of DBC among this cohort of compensated cirrhotics is shown in **Table 4**; univariate analysis demonstrates that older age (OR=1.04), presence of type 2/3 papilla (OR=4.07, OR=3.08), presence of duodenal diverticulum (OR=2.52) and biliary cannulation through precut sphincterotomy (OR=31.25) are significantly associated with difficult cannulation and these were reconfirmed in multivariate analysis.

Difficult biliary cannulation represented by terms of cannulation time and cannulation attempts is a reliable predictor for the occurrence of complications among this cohort of cirrhotics **(Table 5).** At a cut off ≥8.5 min cannulation time yields sensitivity of 80% and specificity of 75.9%, while cannulation attempts ≥4 yield a sensitivity of 86.7% and specificity of 67.9% in predicting ERCP complications in this cohort of compensated cirrhotics.

DISCUSSION

Failed biliary cannulation occurs in up to 20% of ERCP cases and itself is associated with a higher risk of complications including PEP, bleeding, delayed therapy, and others [13]. In the current study failed cannulation occurred in 3.01% of cases, however those patients were treated either through other procedure (n=1) or another ERCP session (n=3). This low frequency of failed cannulation may be related to the small number of patients in the current study in one hand and to improved skills and introduction of assisted canulation techniques in the other hand.

**Estela et al.** [14], reported DBC among 43.9% of 230 patients with naive papilla experiencing their first time ERCP but without figuring out their underlying comorbidities, when compared to 45.5% (55/121) DBC rate in the current study we can infer that naive papilla cannulation is challenging whatever the underlying comorbidity is.

In the current study, mean age of studied cases is 51.86±11.98 years with males representing 51.2%. This mean age is similar to other studies focusing cirrhotics [8,15] and less than other studies focusing on the general patients [16-18], and this may be due to proposed reduced life expectancy among cirrhotics, keeping in mind that a great proportion of our patients had other major co-morbidities. Older age in the current study was associated with DBC and adverse events, this looks inconsistent with previous reports, although other reports [18] does not show age to increase the risk of ERCP complications nor associated with DBC [16], this difference can be explained by the underlying cirrhosis with other commodities in the current study compared to the other studies. Furthermore, the indications of ERCP in the current study were not different from those in the general patients where **Saito et al.** [17]**,** and **Estela et al.** [14], reported CBD stones as the most frequent indication for ERCP in the general patients. Earlier reports [8,15] proposed that patients with underlying liver cirrhosis may have higher frequency of gall stone disease compared to the general patients, and this was confirmed in the current study because 81.9% of patients had been endoscoped because of the calcular obstructive jaundice.

**Tabak et al.,** proposed that DBC among patients with predominant cholelithiasis is associated with increased risk of adverse events [16], while **Fugazza et al.** [16], found that DBC especially with the use of [papillotomy](https://www.sciencedirect.com/topics/medicine-and-dentistry/vater-papillotomy) or the combination of two or more techniques for cannulation was associated with ERCP complications among patients with malignant biliary obstruction and this is re-emphasized in the current study, because all complications were reported in the DBC group. ERCP is a high-risk procedure with 5%–10% of mild-to-severe complications, such as pancreatitis, bleeding, perforation, and infection. Pancreatitis is the most common complication, but various studies showed different frequencies of sequels, which may be due to discrepancy in ERCP difficulty or target populations. Numerous physician, patient, and procedure-based risk factors are related to post-ERCP complications [19,20]. These were re-confirmed in this study; our cohort of cirrhotic patients had 12.6% adverse events rate, a higher-than-average risk patients [17-20], but consistent with previous reports from the same subgroup of cirrhotic patients [8,15,21]. But one question pops up herein, how DBC predispose to ERCP complications, and the answer can be explained based on the prolonged maneuver time spent in attempting cannulation, and the assisted cannulation techniques used e.g., precut sphincterotomy, fistulotomy, double guidewire through pancreatic duct cannulation, all were associated with ERCP complications in the current study **(Table 2)** and in many other studies [17-20]. These techniques exert direct trauma to pancreatic duct enhancing the chance of PEP, and the associated cutting in this vulnerable cohort of cirrhotics who intrinsically had bleeding tendency [8] explain the bleeding reported in the current study. In the present study, duration of the procedure represented as cannulation time (mins) and cannulation attempts can predict the occurrence of ERCP complications in the context of DBC with a cutoff ≥8.5 min for cannulation time yielding sensitivity 80% and specificity 75.9%, and for cannulation attempts ≥4 yielding sensitivity 86.7% and specificity 67.9%.

Our study showed that incidence of complications among the studied cases were 12.6% with 8.7% pancreatitis and 3.9% bleeding. We are in agreement the literature that, the most common complications of ERCP are pancreatitis, followed by hemorrhage [22]. In disagreement with our study, **Chen et al.** [23]**,** included 286 patients who experienced therapeutic ERCP with naïve major duodenal papilla. They noted a complications rate of 6.64% and 4.20% for pancreatitis and bleeding respectively [23], these may be due to the small number of patients in the current study, and the presence of cirrhosis that is a known risk factor for both complications [8,15].

In the current study different comorbidities have been reported and these might impact not only the wellbeing of the patients’ but also the ERCP outcomes, although not the ease of biliary cannulation but obviously the complications. Obesity, nephropathy, lupus and smoking were associated with complications in the current study. **Coelho‐Prabhu et al.** [24], found that BMI ≥35 is a risk factor for post‐ERCP complications, while **Chen et al.** [24], found that morbid obesity and not obesity is associated with increased mortality, length of stay, and total cost in patients undergoing ERCP. **Tabak et al.** [16], proposed that presence of comorbidity index ≥2 is associated with increased risk of adverse events, a reasonable number of our patients had ≥2 commodities **(Table 1)** and this may in part explain the high ERCP complication rate in the current study.

The shape of the duodenal major papilla has been associated not only with DBC but also the rate and type of adverse events. In the current study type 1 papilla was the most common in agreement with other studies [14,17,26]. In the current study type 2 and 3 papilla are associated with DBC, this agrees **Haraldsson et al.** [10]**,** and **Estela et al.** [14]**,** who reported type 2 and type 3 papilla to be associated with DBC respectively.

Furthermore, type 2 papilla is significantly associated with post-ERCP complications as reported earlier by **Wilmer Gustavo et al.** [26]**,** however in the current study type 3 papilla is significantly associated with bleeding **(Table 2)**, probably the large protruding type 3 papilla is associated with more cutting techniques e.g., generous sphincterotomy, in agreement with our findings, **Watanabe et al.** [27], reported that papillary protrusion is an independent risk factor for DBC. While type 2 papilla was non-significantly associated with PEP as reported earlier by **Wilmer Gustavo et al.** [26]. In addition, the cannulation time and cannulation attempts required for cannulating these difficut papillae (type 2 and 3) were predictors for complications in the current study and its validities for predicting complications were 80 and 86% repectively, results that agrees previous notices of **Haraldsson et al.** [10]***,*** who found that type 2 and 3 papi­llae were more difficult to cannulate (OR = 1.89 and 1.61, respectively) compared to type 1 and 4 papillae. In addi­tion, the median cannulation time was significantly longer for type 2 papilla (269 seconds) and type 3 papilla (245 seconds), both with a *p* < 0.05 value, compared to type 1 papilla (139 seconds).

In the current study, univariate analysis for predictors of DBC demonstrates that the following are significantly associated with difficult cannulation; older age (OR=1.04), presence of type 2 (OR=4.07) and 3 papilla (OR=3.08), presence of duodenal diverticulum (OR=2.52) and precut sphincterotomy (OR=31.25). We can notice some agreement with different publications, where **Ben Abdallah et al.** [28], found that small papilla (type 2 in Haraldsson classification), **and Saito et al,** found the presence of diverticulum, were predictable of DBC, although both publications found trainee/non-experienced endoscopists incorporation is a predictive factor for DBC, unfortunately this was not studied in the current study. We did not find evidence in the literature specifying older age as a predicting factor for DBC, although with advancing age the frequency of duodenal diverticula may be increased, and this might favor DBC in this sub-group of patients.

This study had its own limitations that are not limited to small number of patients recruited but also include some others. Being a single center study impairs its reliability. Lack of studying the impact of operator related factors such as endoscopist lever of expertise and trainee involvement and some other procedure related factors e.g., stenting, balloon dilation, in the DBC chances. Lack of a direct comparison between patients with cirrhosis and patients without cirrhosis. Comparing cirrhotic to non-cirrhotic patients will highlight the challenges unique to the event of cirrhosis regarding the difficulty of cannulation, predictors of difficult cannulation in relation to cirrhosis, and also adverse events peculiar to cirrhotics. In addition, exclusion of patients with decompensated cirrhosis who represent real challenge we daily face in our practice. Furthermore, the long-term impact of ERCP procedure and the resultant complications on the natural history of the current cohort of compensated cirrhotics was not focused on. Hopefully, future studies can cover these limitations.

**CONCLUSION**

DBC was reported among 45.5% of compensated cirrhotic patients experiencing their first time ERCP due to different indications mainly cholelithiasis. DBC was associated with ERCP related complications. All complications including pancreatitis and intraoperative bleeding were reported in DBC group. Predictors of DBC include older age (OR=1.04), presence of type 2 (OR=4.07) and 3 papillae (OR=3.08), presence of duodenal diverticulum (OR=2.52) and precut sphincterotomy (OR=31.25). Although the current study results are different from what is actually known, yet it confirms earlier results and opens the door to further investigate these sub-group of patients who are seen in daily practice.

**DECLARATIONS**

● Abbreviations: Endoscopic retrograde cholangiopancreatography; ERCP, magnetic resonance imaging; MRI, magnetic resonance cholangiopancreatography; MRCP, endoscopic ultrasound; EUS, difficult biliary cannulation; DBC, post-ERCP pancreatitis; PEP.

● Ethics approval and consent to participate:

This study was carried out in concordance with sound medical practice and according to declaration of Helsinki. Approval to carry out the study was obtained from the IRB committee of the Faculty of Medicine, Kafrelsheikh University, Egypt (KFSIRB200-480) and all patients signed an informed consent after explanation about the concept, design, benefits and risks of the study

● Consent for publication: Obtained

● Availability of data and materials: The datasets used and/or analysed during the current study are available from the corresponding author on reasonable request.

● Competing Interests: None

● Funding: None. Author funded

● Authors' contributions: Elkerdawy M, Mahros A, Elbatae H, Ahmed M; research design, Elkerdawy M, Mahros A, Ahmed M, Ebeed A, Emara M, acquisition, analysis or interpretation of data; Elkerdawy M, Emara M drafted the paper, Elbtae H, Mahros A, Ahmed M revised it critically. All authors approved the submitted version.

● Acknowledgements: The authors would thank all colleagues who helped to conduct this study.

**REFERENCES:**

1. ASGE Standards of Practice Committee; Chandrasekhara V, Khashab MA, Muthusamy VR, Acosta RD, Agrawal D, et al. Adverse events associated with ERCP. Gastrointest Endosc. 2017 Jan;85(1):32-47.
2. ASGE Standards of Practice Committee; Chathadi KV, Chandrasekhara V, Acosta RD, Decker GA, Early DS, et al. The role of ERCP in benign diseases of the biliary tract. Gastrointest Endosc. 2015 Apr;81(4):795-803.
3. ASGE Standards of Practice Committee; Early DS, Ben-Menachem T, Decker GA, Evans JA, Fanelli RD, et al. Appropriate use of GI endoscopy. Gastrointest Endosc. 2012 Jun;75(6):1127-31.
4. Johnson KD, Perisetti A, Tharian B, Thandassery R, Jamidar P, Goyal H, et al. Endoscopic Retrograde Cholangiopancreatography-Related Complications and Their Management Strategies: A "Scoping" Literature Review. Dig Dis Sci. 2020 Feb;65(2):361-375.
5. Han S, Zhang J, Durkalski-Mauldin V, Foster LD, Serrano J, Coté GA, et al. Impact of difficult biliary cannulation on post-ERCP pancreatitis: secondary analysis of the stent versus indomethacin trial dataset. Gastrointest Endosc. 2024 Oct 9:S0016-5107(24)03548-X.
6. Fugazza A, Troncone E, Amato A, Tarantino I, Iannone A, Donato G, et al. Difficult biliary cannulation in patients with distal malignant biliary obstruction: An underestimated problem? Dig Liver Dis. 2022 Apr;54(4):529-536.
7. Andriulli A, Loperfido S, Napolitano G, Niro G, Valvano MR, Spirito F, et al. Incidence rates of post-ERCP complications: a systematic survey of prospective studies. Am J Gastroenterol. 2007 Aug;102(8):1781-8.
8. Emara MH, Elbatee HE, Radwan MI, Amer IF, Zaghloul MS, Ahmed MA et al. ERCP among Cirrhotics: What are the differences?". Afro-Egypt J Infect Endem Dis. 2020;10(3): 241-248.
9. Malik R, Mookerjee RP, Jalan R. Infection and inflammation in liver failure: two sides of the same coin. J Hepatol. 2009 Sep;51(3):426-9.
10. Haraldsson E, Kylänpää L, Grönroos J, Saarela A, Toth E, Qvigstad G, et al. Macroscopic appearance of the major duodenal papilla influences bile duct cannulation: a prospective multicenter study by the Scandinavian Association for Digestive Endoscopy Study Group for ERCP. Gastrointest Endosc. 2019 Dec;90(6):957-963.
11. Testoni PA, Mariani A, Aabakken L, Arvanitakis M, Bories E, Costamagna G, et al. Papillary cannulation and sphincterotomy techniques at ERCP: European Society of Gastrointestinal Endoscopy (ESGE) Clinical Guideline. Endoscopy. 2016 Jul;48(7):657-83.
12. Banks PA, Bollen TL, Dervenis C, Gooszen HG, Johnson CD, Sarr MG, et al. Acute Pancreatitis Classification Working Group. Classification of acute pancreatitis--2012: revision of the Atlanta classification and definitions by international consensus. Gut. 2013 Jan;62(1):102-11.
13. Berry R, Han JY, Tabibian JH. Difficult biliary cannulation: Historical perspective, practical updates, and guide for the endoscopist. World J Gastrointest Endosc. 2019 Jan 16;11(1):5-21.
14. Estela EL, Tovar NR, Maldonado FA, Tisoc LM, Goicochea-Lugo S, Rossell MC. Association between type of major duodenal papilla and difficult biliary cannulation at first endoscopic retrograde cholangiopancreatography in adults: a cross-sectional study with bootstrap method. Ann Gastroenterol. 2023 Mar-Apr;36(2):216-222.
15. El-Naggar YA, Radwan MI, Emara MH. Needle-Knife Fistulotomy as a Rescue Access of the Common Bile Duct in Cirrhotic Patients: Feasibility and Outcome. Afro-Egypt J Infect Endem Dis. 2013, 3(4): 135-145.
16. Tabak F, Wang HS, Li QP, Ge XX, Wang F, Ji GZ, Miao L. Endoscopic retrograde cholangiopancreatography in elderly patients: Difficult cannulation and adverse events. World J Clin Cases. 2020 Jul 26;8(14):2988-2999.
17. Saito H, Kadono Y, Shono T, Kamikawa K, Urata A, Nasu J, Imamura H, Matsushita I, Kakuma T, Tada S. Factors Predicting Difficult Biliary Cannulation during Endoscopic Retrograde Cholangiopancreatography for Common Bile Duct Stones. Clin Endosc. 2022 Mar;55(2):263-269.
18. Zuleta MG, Melgar C, Arbeláez V. Does age influence complications of endoscopic retrograde cholangiopancreatography (ERCP)?. Rev Col Gastroenterol 2010; 25(4):347-351.
19. Fujisawa T, Kagawa K, Hisatomi K, Kubota K, Nakajima A, Matsuhashi N. Is endoscopic papillary balloon dilatation really a risk factor for post-ERCP pancreatitis? World J Gastroenterol. 2016 Jul 14;22(26):5909-16.
20. Szary NM, Al-Kawas FH. Complications of endoscopic retrograde cholangiopancreatography: how to avoid and manage them. Gastroenterol Hepatol (N Y). 2013 Aug;9(8):496-504.
21. Attia, A. Safety, risk stratification, and cost of ERCP in patients with cirrhosis: a prospective controlled study. Egypt Liver Journal 14, 7 (2024). <https://doi.org/10.1186/s43066-024-00311-0>)
22. Andriulli A, Loperfido S, Napolitano G, Niro G, Valvano MR, Spirito F, Pilotto A, Forlano R. Incidence rates of post-ERCP complications: a systematic survey of prospective studies. Am J Gastroenterol. 2007 Aug;102(8):1781-8.
23. Chen PH, Tung CF, Peng YC, Yeh HZ, Chang CS, Chen CC. Duodenal major papilla morphology can affect biliary cannulation and complications during ERCP, an observational study. BMC Gastroenterol. 2020 Sep 23;20(1):310.
24. Coelho‐Prabhu N, Shah ND, Van Houten H, Kamath PS, Baron TH. Endoscopic retrograde cholangiopancreatography: utilisation and outcomes in a 10‐year population‐based cohort. BMJ Open. 2013;3:e002689.
25. Chen B, Yo CH, Patel R, Liu B, Su KY, Hsu WT, Lee CC. Morbid obesity but not obesity is associated with increased mortality in patients undergoing endoscopic retrograde cholangiopancreatography: A national cohort study. United European Gastroenterol J. 2021 Jun;9(5):561-570.
26. Wilmer Gustavo Q-P W, Ricardo P-A D, Amparo B-P J, Andrei V-B D. Morphological Characteristics of the Duodenal Papilla and its Association with Complications Post-Endoscopic Retrograde Cholangiopancreatography (ERCP) in a Peruvian Hospital. Rev. colomb. Gastroenterol. 2022; 37(3): 296-301.
27. Watanabe M, Okuwaki K, Kida M, Imaizumi H, Yamauchi H, Kaneko T, Iwai T, Hasegawa R, Miyata E, Masutani H, Tadehara M, Adachi K, Koizumi W. Transpapillary Biliary Cannulation is Difficult in Cases with Large Oral Protrusion of the Duodenal Papilla. Dig Dis Sci. 2019 Aug;64(8):2291-2299.
28. Ben Abdallah K, Hamzaoui L, Mahmoudi M, Cherif I, Ben Mohamed A, Yakoubi M, Khsiba A, Medhioub M, Azouz MM. Predictive factors of difficult biliary cannulation: An experience of a tunisian tertiary center. Heliyon. 2022 Dec 22;8(12):e12526.

**Figure legends:**

**Figure 1:** Study Flow Chart


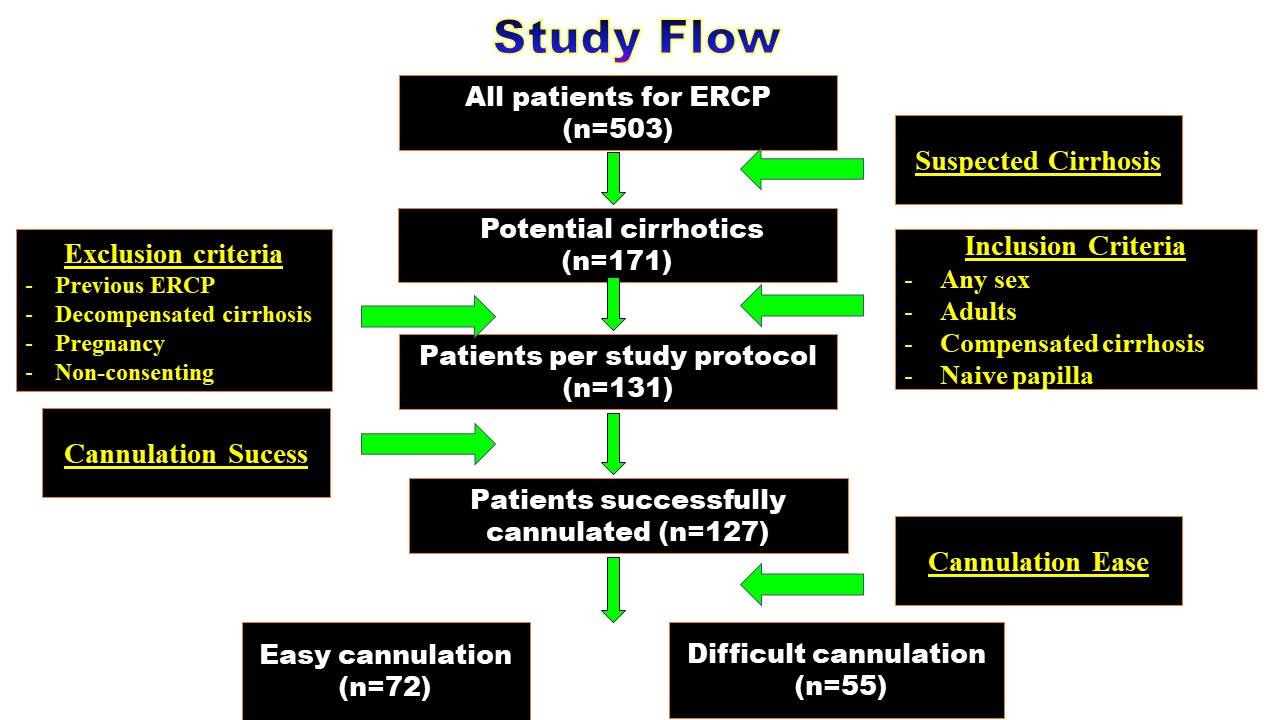


**Table (1): Demographic and Clinical data of the studied groups**

|  | **Difficult cannulation** | | **Test of significance** | **Complication** | | **Test of significance** |
| --- | --- | --- | --- | --- | --- | --- |
|  | **No**  **n=72** | **Yes**  **n=55** |  | **No**  **n=112** | **Yes**  **n=15** |  |
| **Demographic characters** | | | | | | |
| Age / years | 49.58±10.28 | 54.84±13.42 | t=2.49  P=0.014* | 50.61±11.92 | 61.2±7.64 | t=3.34  P=0.001* |
| Sex  Male  Female | 39(54.2)  22(45.8) | 26(47.3)  29(65.7) | ꭓ^2^=0.593  p=0.441 | 55(49.1)  57(50.9) | 10(66.7)  5(33.3) | ꭓ^2^=1.63  p=0.201 |
| **Associated comorbidities** | | | | | | |
| Diabetes | 19(26.4) | 20(36.4) | ꭓ^2^=1.46  p=0.227 | 32(28.6) | 7(46.7) | ꭓ^2^=2.04  p=0.154 |
| Hypertension | 19(26.4) | 16(29.1) | ꭓ^2^=0.114  p=0.736 | 28(25) | 7(46.7) | ꭓ^2^=3.11  p=0.08 |
| Smoking | 19(26.4) | 13(23.6) | ꭓ^2^=0.125  p=0.723 | 25(22.3) | 7(46.7) | ꭓ^2^=4.16  p=0.04* |
| Other comorbidities | 5(6.9) | 10(18.2) | ꭓ^2^=3.78  p=0.093 | 10(8.9) | 5(33.3) | ꭓ^2^=7.56  p=0.006* |
| Surgical history | 3(4.2) | 3(5.5) | FET=0.115  P=1.0 | 6(5.4) | 0 | ꭓ^2^=0.843  p=0.358 |
| **Indication and clinical presentation** | | | | | | |
| Clacular Obstructive jaundice | 62(86.1) | 42(76.4) | ꭓ^2^=1.99  p=0.158 | 93(93) | 11(73.3) | ꭓ^2^=0.840  p=0.358 |
| Biliary pain | 8(11.1) | 11(20) | ꭓ^2^=1.94  p=0.164 | 15(13.4) | 4(26.7) | ꭓ^2^=1.83  p=0.176 |
| Cholangitis | 6(8.3) | 4(7.3) | ꭓ^2^=0.048  p=0.826 | 10(8.9) | 0 | ꭓ^2^=1.45  p=0.228 |
| Cancer | 7(9.7) | 9(16.4) | ꭓ^2^=1.25  p=0.264 | 13(11.6) | 3(20.0) | ꭓ^2^=0.846  p=0.358 |
| Pancreatitis | 1(1.4) | 1(1.8) | FET=0.037  P=1.0 | 2 (1.8) | 0 | ꭓ^2^=0.272  p=1.0 |

t: Student t test ꭓ^2^=Chi-Square test , FET: Fisher exact test *statistically significant

**Table (2): Endoscopic data in relation to difficult cannulation and complications among studied cases.**

|  | Difficult cannulation | | Test of significance | Complication | | Test of significance |
| --- | --- | --- | --- | --- | --- | --- |
|  | No  N=72(%) | Yes  N=55(%) |  | No  N=112 | Yes  N=15 |  |
| CBD  Dilated with  Distal Stricture | 17(23.6)  55(76.4) | 28(50.9)  27(49.1) | ꭓ^2^=10.16  P=0.001* | 38(33.9)  74(66.1) | 7(46.7)  8(53.3) | ꭓ^2^=0.938  P=0.333 |
| Size of dilated CBD (mm) | 12.39±2.65 | 11.8±3.39 | t=1.09  p=0.278 | 12.12±2.94 | 12.2±3.59 | t=0.097  p=0.923 |
| Distal stones size /mm | 6.56±2.56 | 7.37±2.67 | t=1.32  p=0.191 | 6.73±2.58 | 7.75±2.87 | t=1.05  p=0.297 |
| Pancreatic masses | 7(9.7) | 9(16.4) | ꭓ^2^=1.25  P=0.291 | 13(11.6) | 3(20) | ꭓ^2^=0.846  P=0.358 |
| Shape of papilla  Type1  Type2  Type3  Type4 | 50(69.4)  5(6.9)  6(8.3)  11(15.3) | 27(49.1)  11(20)  10(18.2)  7(12.7) | ꭓ^2^=8.89  P=0.03* | 73(65.2)  11(9.8)  13(11.6)  15(13.4) | 4(26.7)  5(33.3)  3(20)  3(20) | ꭓ^2^=10.19  P=0.017* |
| Presence of diverticulum | 16(22.2) | 23(41.8) | ꭓ^2^=5.62  P=0.018* | 31(27.7) | 8(53.3) | ꭓ^2^=4.09  P=0.04* |
| Cannulation technique | | | | | | |
| Guidewire assisted | 69(95.8) | 6(10.9) | ꭓ^2^=93  P=0.001* | 75(67) | 0 | ꭓ^2^=24.53  P=0.001* |
| Precut sphincterotomy | 3(4.2) | 39(70.9) | ꭓ^2^=62.75  P=0.001* | 33(29.5) | 9(60.0) | ꭓ^2^=5.57  P=0.018* |
| Double guide | 0 | 6(10.9) | ꭓ^2^=14.21  P=0.001* | 2(1.8) | 4(26.7) | ꭓ^2^=24.84  P=0.001* |
| Fistulotomy | 0 | 4(7.3) | ꭓ^2^=5.41  P=0.02* | 2(1.8) | 2(13.3) | ꭓ^2^=5.78  P=0.068 |
| Cannulation time (mins) | 4(2-5) | 9(4-23) | Z=9.59  P=0.001* | 5(2-23) | 11(8-19) | Z=4.67  P=0.001* |
| Cannulation attempts | 2(1-5) | 4(2-8) | Z=8.82  P=0.001* | 2(1-6) | 5(3-8) | Z=4.56  P=0.001* |

CBD= common bile duct, ꭓ^2^=Chi-Square test, FET: Fisher exact test *statistically significant# include IHBR and cirrhosis

**Table (3): Frequency of complications among studied cases**

| **Complications** | **Difficult cannulation** | | | | | | **p-value** | |
| --- | --- | --- | --- | --- | --- | --- | --- | --- |
|  | **No**  **N=72** | | | **Yes**  **N=55** | | |  | |
| **Pancreatitis** | 0 | | | 11(20%) | | | P<0.001* | |
| **Bleeding** | 0 | | | 5(9.1%) | | | P=0.014* | |
| **Infection** | 0 | | | 0 | | | … | |
| **Perforation** | 0 | | | 0 | | | … | |
| **Death** | 0 | | | 0 | | | … | |
|  | **Shape of papilla** | | | | | | **P value** | |
|  |  | 1 | 2 | | 3 | 4 |  |  |
| **Bleeding** | N | 0 | 1 | | 2 | 2 | 0.03* |  |
|  | % | .0% | 6.2% | | 12.5% | 11.1% |  |  |
| **Pancreatitis** | N | 4 | 4 | | 1 | 2 | 0.08 |  |
|  | % | 5.2% | 25.0% | | 6.2% | 11.1% |  |  |
| Mild | 0 | 4 | 2 | | 0 | 2 |  |  |
| Moderate | 0 | 0 | 1 | | 0 | 0 |  |  |
| severe | 0 | 0 | 1 | | 1 | 0 |  |  |

ꭓ^2^=Chi-Square test , *statistically significant

**Table (4): Univariate and multivariate analysis for predictors of difficult cannulation among studied cases**

|  | Univariate analysis | | | Multivariate analysis | |
| --- | --- | --- | --- | --- | --- |
|  | Β | P value | Odds ratio  (95% CI) | P value | Odds ratio  (95% CI) |
| Age / years | 0.039 | 0.016* | 1.04  (1.01-1.07) | 0.03* | 1.03  (1.01-1.09) |
| Shape of papilla  Type1  Type2  Type3  Type4 | r  1.405  1.127  .164 | .039*  .017*  .048*  .761 | R  4.07(1.28-12.94)  3.08(1.01-9.41)  1.18(0.41-3.39) | 0.03*  0.06  0.821 | R  3.1(2.56-6.9)  3.12(0.89-9.6)  1.15(0.32-5.6) |
| Presence of diverticulum | 0.923 | 0.019* | 2.52(1.16-5.44) | 0.04* | 1.56(1.25-6.8) |
| Precut sphincterotomy | 3.59 | 0.001* | 36.29(8.08-162.94) | 0.002* | 31.25(7.69-160.8) |
| Guidewire assisted | -4.93 | 0.051 | 0.007(0.002-2.027) | 0.19 | 0.004(0.001-3.08) |

β: regression coefficient , *statistically significant, CI: Confidence interval

**Table (5): validity of Cannulation time (mins), Cannulation attempts in prediction of complications**

| Test Result Variable(s) | Area | Std. Error^a^ | P VALUE | Asymptotic 95% Confidence Interval | | Cut off points | Sensitivity | Specificity |
| --- | --- | --- | --- | --- | --- | --- | --- | --- |
|  |  |  |  | Lower Bound | Upper Bound |  |  |  |
| Cannulation time (mins) | .865 | .034 | .001* | .798 | .933 | ≥8.5 | 80.0 | 75.9 |
| Cannulation attempts | .855 | .043 | .001* | .771 | .939 | ≥4 | 86.7 | 67.9 |
